# BET protein inhibitor apabetalone (RVX-208) suppresses pro-inflammatory hyper-activation of monocytes from patients with cardiovascular disease and type 2 diabetes

**DOI:** 10.1186/s13148-020-00943-0

**Published:** 2020-11-11

**Authors:** Sylwia Wasiak, Kim E. Dzobo, Brooke D. Rakai, Yannick Kaiser, Miranda Versloot, Mahnoush Bahjat, Stephanie C. Stotz, Li Fu, Michael Sweeney, Jan O. Johansson, Norman C. W. Wong, Erik S. G. Stroes, Jeffrey Kroon, Ewelina Kulikowski

**Affiliations:** 1 Resverlogix Corp, 300-4820 Richard Road SW, Calgary, AB T3E 6L1 Canada; 2 Department of Experimental Vascular Medicine, Amsterdam Cardiovascular Sciences, Amsterdam UMC, University of Amsterdam, Meibergdreef 9, Amsterdam, The Netherlands; 3 Department of Vascular Medicine, Amsterdam Cardiovascular Sciences, Amsterdam UMC, University of Amsterdam, Meibergdreef 9, Amsterdam, The Netherlands

**Keywords:** Bromodomain, Transcription regulation, Innate immune response, Cardiovascular, Apabetalone

## Abstract

**Background:**

Patients with cardiovascular disease (CVD) and type 2 diabetes (DM2) have a high residual risk for experiencing a major adverse cardiac event. Dysregulation of epigenetic mechanisms of gene transcription in innate immune cells contributes to CVD development but is currently not targeted by therapies. Apabetalone (RVX-208) is a small molecule inhibitor of bromodomain and extra-terminal (BET) proteins—histone acetylation readers that drive pro-inflammatory and pro-atherosclerotic gene transcription. Here, we assess the impact of apabetalone on ex vivo inflammatory responses of monocytes from DM2 + CVD patients.

**Results:**

Monocytes isolated from DM2 + CVD patients and matched controls were treated ex vivo with apabetalone, interferon γ (IFNγ), IFNγ + apabetalone or vehicle and phenotyped for gene expression and protein secretion. Unstimulated DM2 + CVD monocytes had higher baseline IL-1α, IL-1β and IL-8 cytokine gene expression and Toll-like receptor (TLR) 2 surface abundance than control monocytes, indicating pro-inflammatory activation. Further, DM2 + CVD monocytes were hyper-responsive to stimulation with IFNγ, upregulating genes within cytokine and NF-κB pathways > 30% more than control monocytes (*p* < 0.05). Ex vivo apabetalone treatment countered cytokine secretion by DM2 + CVD monocytes at baseline (GROα and IL-8) and during IFNγ stimulation (IL-1β and TNFα). Apabetalone abolished pro-inflammatory hyper-activation by reducing TLR and cytokine gene signatures more robustly in DM2 + CVD versus control monocytes.

**Conclusions:**

Monocytes isolated from DM2 + CVD patients receiving standard of care therapies are in a hyper-inflammatory state and hyperactive upon IFNγ stimulation. Apabetalone treatment diminishes this pro-inflammatory phenotype, providing mechanistic insight into how BET protein inhibition may reduce CVD risk in DM2 patients.

## Background

Low-density lipoprotein cholesterol (LDL-C) lowering is the key strategy to prevent cardiovascular disease (CVD). However, even a substantial reduction in LDL-C still leaves patients at a significant residual risk of cardiovascular adverse events. Amongst others, type 2 diabetes mellitus (DM2) is recognized as a contributor to CVD risk [1]. This comorbidity is likely caused by organ exposure to chronic systemic low-grade inflammation caused by elevated circulating levels of cytokines IL-6 and IL-1β, glucose, free fatty acids and reactive oxygen species (ROS) [2–6]. Such a microenvironment favours monocyte infiltration into the arterial wall, where these cells differentiate into macrophages that contribute to initiation and progression of atherosclerosis. Ultimately, macrophage activity in atherosclerotic plaque precipitates atherothrombosis and clinical cardiovascular events [7].

Monocyte activity is not restricted to the arterial wall as circulating monocytes are also a major source of pro-inflammatory and pro-oxidant factors [7]. In fact, monocytes from patients with advanced atherosclerosis and/or hypercholesterolemia are hyper-responsive and therefore produce more pro-inflammatory cytokines, such as interleukin (IL) 6, IL-1β and TNFα [8–10]. This pro-inflammatory state is partially ascribed to hypercholesterolemia, which reprograms myeloid progenitors in the bone marrow to produce hyperactive monocytes and macrophages in experimental atherosclerosis models [11–13]. The resulting “immunological memory” is encoded by epigenetic changes to chromatin in the form of DNA methylation and histone post-translational modification [14]. The contribution of histone marks to persistent immune cell activation has been demonstrated in atherosclerotic mouse models [15, 16], in human monocytes isolated from DM2 and CVD patients’ blood or from atherosclerotic plaques [9, 10, 17–19]. This evidence of widespread epigenetic dysregulation in activated immune cells opens the window for epigenetic regulators as therapeutic agents in DM2 and CVD [14].

Bromodomain and extraterminal (BET) proteins (BRD2, BRD3, BRD4 and BRDT) are histone acetylation “readers” generally linked to the induction of gene transcription [20]. BET proteins are recruited to gene enhancers and promoters via direct binding to acetylated chromatin or to acetylated transcription factors, such as the nuclear factor κ-light-chain-enhancer of activated B cells (NF-κB) and the signal transducer and activator of transcription (STAT) [21–25]. Once chromatin-bound, BETs recruit chromatin remodeling and transcription elongation factors, leading to activation of RNA polymerase II and transcription of proximal genes [26]. Since BET proteins play a critical role in transcription of cytokine response genes involved in inflammation, lipid metabolism and vascular function [23, 27–30], inhibiting their activity could prove beneficial for the treatment of chronic inflammatory and metabolic diseases.

Apabetalone (RVX-208) is an orally available small molecule BET inhibitor (BETi) that mimics the endogenous ligand of BET proteins, the acetylated lysine residue. Apabetalone preferentially binds to the second of two conserved BET protein bromodomains (with > 20-fold higher affinity) [31–33], countering BET protein recruitment to chromatin. Consequently, apabetalone inhibits transcription of BET-dependent genes [31, 34]. In vitro treatment with apabetalone reduces pro-inflammatory gene expression in cellular models of atherosclerosis, including endothelial cells [34, 35], monocytes [34] and vascular smooth muscle cells [36]. Apabetalone also reduces vascular inflammation and atherosclerosis in mouse models [23, 35]. These data suggest that apabetalone could correct the pro-inflammatory phenotype of innate immune cells characteristic of DM2 and CVD.

Here, we demonstrate that monocytes isolated from patients with DM2 and CVD (DM2 + CVD) have an enhanced pro-inflammatory phenotype as compared to matched controls. Moreover, challenging DM2 + CVD monocytes ex vivo with interferon gamma (IFNγ), a key cytokine that triggers monocyte differentiation into the pro-inflammatory macrophage M1 subtype [37], provokes a hyperactive transcriptional response as compared to controls. Ex vivo apabetalone treatment diminishes this hyper-inflammatory state, suggesting that BET protein inhibition can mitigate monocyte-driven inflammation in patients with high residual risk for major adverse cardiovascular events.

## Results

### Apabetalone suppresses pro-inflammatory cytokine secretion in monocytes from DM2 + CVD patients

For this study, we recruited patients with DM2 and stable CVD (DM2 + CVD) (*n* = 14), and age- and gender-matched control subjects (*n* = 12) (Table 1). DM2 + CVD patients, on standard of care including insulin and/or statins, had higher glucose levels (mean 8.05, [7.30–10.15]) than controls (mean 5.35, [5.20–5.60]). They also had increased systolic blood pressure (142.93 (13.38)), elevated triglycerides levels (1.39, [1.13–1.70]), and reduced HDL levels (1.31 (0.22)). Control subjects were not on any medication.

**Table 1:** Baseline clinical characteristics of enrolled subjects

Clinical characteristics	DM2 + CVD (*n* = 14)	Controls (*n* = 12)	*p* value
Age	68.39 (5.08)	68.09 (5.16)	0.9
Gender (male %)	7 (50)	8 (66.7)	0.7
BMI (mean (SD))	31.06 (6.85)	26.71 (5.66)	0.09
Lifestyle			
Current smoker	0 (0)	0 (0)	1
Former smoker	7 (50)	6 (50.0)	1
Past smoker	7 (50)	6 (50.0)	1
Pack-years (mean (SD))	9.50 [0–45]	4.00 [0–10.5]	0.06
Systolic BP (mean (SD))	142.93 (13.38)	127.5 (15.21)	0.01
Medical history			
AP (%)	7 (50)	0 (0)	0.02
MI (%)	4 (28.6)	0 (0)	0.1
CVA (%)	2 (14.3)	0 (0)	0.5
PAD (%)	6 (42.9)	0 (0)	0.03
CRP (median [IQR])	1.50 [1.30–3.15]	1.35 [0.85–2.02]	0.3
Leukocytes (mean (SD))	6.59 (1.35)	5.57 (1.65)	0.1
Monocytes (mean (SD))	0.56 (0.17)	0.50 (0.13)	0.4
Glucose (median [IQR])	8,05 [7.30–10.15]	5.35 [5.20–5.60]	< 0.001
Creatinine (median [IQR])	89.5 [84–113.75]	84.5 [66.5–92]	0.08
Total cholesterol (mean (SD))	3.97 (0.77)	5.48 (1.21)	0.001
HDL cholesterol (mean (SD))	1.31 (0.22)	1.73 (0.69)	0.04
LDL cholesterol (mean (SD))	1.93 (0.47)	3.23 (0.79)	< 0.001
Triglycerides (median [IQR])	1.39 [1.13–1.70]	0.85 [0.71–1.29]	0.02
Lp(a) (median [IQR])	103 [90–120.5]	87.5 [45.25–148.75]	0.9
Hba1c (median [IQR])	63 [54.5–66.75]	38.5 [38–39.25]	< 0.001
Statin use (%)	12 (85.7)	0	< 0.001
Insulin use (%)	10 (71.4)	0	0.001

Data are presented as the mean (SD), median (IQR) or *n* (%)

*BMI* body mass index, *BP *blood pressure, *AP* angina pectoris, *MI* myocardial infarction; *CVA* cerebrovascular accident, *PAD* peripheral arterial disease, *CRP* C-reactive protein, *HDL* high-density lipoprotein, *LDL* low-density lipoprotein; *Lp(a)* lipoprotein (a)

**Fig. 1:**
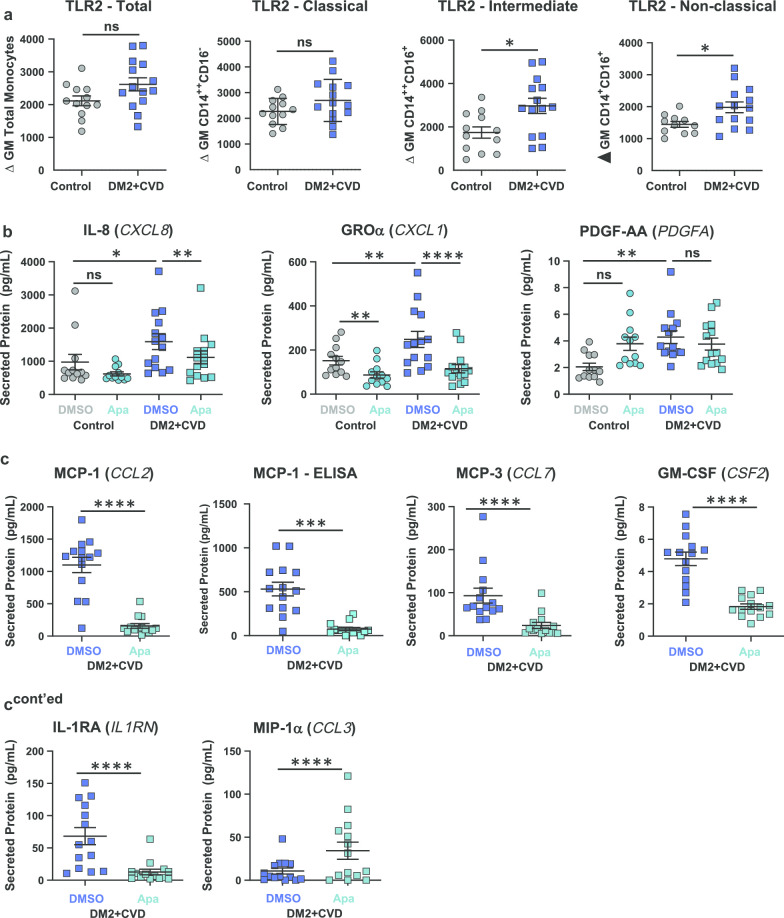
Ex vivo treatment with apabetalone (Apa) abolishes pro-inflammatory protein secretion in monocytes isolated from DM2 + CVD patients. **a** Flow cytometry analysis of the pro-inflammatory surface receptor TLR2 in DM2 + CVD versus control monocytes. **b** Quantification of secreted cytokines 24 h post-plating (Milliplex®) in DMSO or apabetalone (25 μM)-treated control and DM2 + CVD monocytes. **c** Effect of apabetalone (25 μM) treatment on secretion of key pro- and anti-inflammatory cytokines in DM2 + CVD monocytes. Statistics: **a** Unpaired Student’s *t*-test, *p < 0.05; ns, non-significant. **b**, **c** 2-Way repeated measures ANOVA followed by Tukey’s multiple comparisons correction for within-group comparisons, or Bonferroni’s test for between-group comparisons; *p < 0.05; **p < 0.01; ***p < 0.001; ****p < 0.0001. Individual patient data are shown as a mean ± SEM

Monocytes were isolated from whole blood of patients with DM2 + CVD or matched control subjects. Total monocyte number (Table 1) and distribution across subset classifications (Additional file 1) were similar in both cohorts. Profiling of 16 monocyte surface receptors revealed higher expression of Toll-like receptor (TLR) 2 on intermediate (CD14^++^CD16^+^) and non-classical (CD14^+^CD16^+^) DM2 + CVD monocytes (*n* = 14) as compared to controls (*n* = 12) (Fig. 1a), consistent with published observations [38–40]. No change in TLR2 abundance was noted in the total or classical monocyte population (Fig. 1a).

The multianalyte immunoprofiling Milliplex® panel detected 12 cytokines that were secreted by monocytes cultured ex vivo for 24 h. At baseline, monocytes from DM2 + CVD patients (n = 14) showed higher levels of chemokine IL-8 (encoded by the *CXCL8* gene), growth-related oncogene-α (GRO-α, encoded by the *CXCL1* gene) and PDGF-AA, as compared to controls (*n* = 12) (Fig. 1b). Notably, apabetalone (25 μM) lowered the enhanced secretion of IL-8 and GROα in DM2 + CVD monocytes (Fig. 1b). Similarly, apabetalone decreased the secretion of monocyte chemoattractant protein 1 (MCP-1) (also detected by ELISA), MCP-3, granulocyte-macrophage colony stimulating factor (GM-CSF) and interleukin 1 receptor antagonist IL-1RA) (Fig. 1c). Macrophage inflammatory protein MIP-1α (CCL3) was the only protein whose secretion increased with apabetalone treatment (Fig. 1c); no changes were detected in *CCL3* mRNA transcript levels (data not shown). IL-1β, TNFα, IP-10 and RANTES were not differentially secreted at baseline nor affected by apabetalone treatment (not shown). No toxicity was noted across all treatments and time points (Additional file 2). These data indicate that DM2 + CVD monocytes secreted higher levels of pro-inflammatory cytokines ex vivo, which was abolished by treatment with apabetalone.

### Apabetalone abolishes “hyperactive” gene expression in monocytes from DM2 + CVD patients

**Fig. 2:**
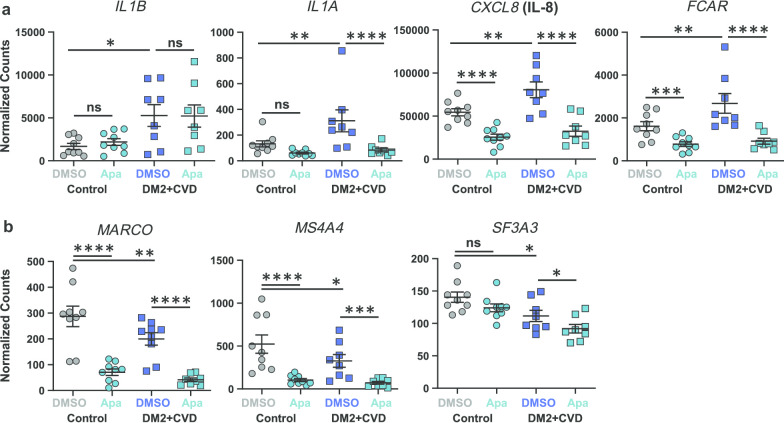
Monocytes from DM2 + CVD patients and control subjects exhibit differential gene expression that is inhibited by 25 μM apabetalone (Apa). **a** Pro-inflammatory genes that show elevated expression in DM2 + CVD monocytes as compared to controls. b. Genes that show reduced expression in DM2 + CVD monocytes as compared to controls. Gene expression is expressed as endogenous control-normalized counts (NanoString™). Statistics: 2-Way repeated measures ANOVA followed by Tukey’s multiple comparisons correction for within-group comparisons, or Bonferroni’s test for between-group comparisons; *p < 0.05; **p < 0.01; ***p < 0.001; ****p < 0.0001; ns, non-significant. Individual patient data are shown as a mean ± SEM

The monocyte gene transcription profile was generated for DM2 + CVD monocytes (*n* = 8) and control monocytes (*n* = 9) using the NanoString nCounter® Vantage 3D™ Innate Immunity Panel (0.025% DMSO, 4 h). Transcripts for 109 of the 180 genes in the panel were detected (endogenous control-normalized counts > 50) in at least one treatment condition. Between-cohort baseline comparison showed that mRNA transcripts encoding pro-inflammatory cytokines (*IL1B, IL1A* and *CXCL8*) and the receptor for the Fc region of IgA (*FCAR*) were more abundant in monocytes from DM2 + CVD patients than controls (Fig. 2a). In contrast, transcripts encoding the macrophage receptor with collagenous structure (*MARCO*), the membrane-spanning 4-domains subfamily A member 4A (*MS4A4A*) and the splicing factor 3A subunit 3 (*SF3A3*) were lower in abundance in monocytes from DM2 + CVD patients. Collectively, these results indicate that the monocytes of DM2 + CVD patients exhibit a hyperactive pro-inflammatory transcriptional state.

To examine whether the hyperactive pro-inflammatory gene transcription can be diminished by BET inhibition, monocytes from patients and control subjects were treated ex vivo with apabetalone (5 or 25 μM) or DMSO (vehicle control) for 4 h. With the exception of *IL1B*, mRNA transcripts overexpressed in DM2 + CVD monocytes (*IL1A*, *CXCL8*, *FCAR*) were downregulated by treatment with 25 μM apabetalone (Fig. 2a). mRNA transcripts expressed at lower levels in DM2 + CVD monocytes versus controls (*MARCO*, *MS4A4A* and *SF3A3*) were further reduced by apabetalone (Fig. 2b). Transcription of *BRD4*, the gene encoding the BRD4 BET protein, was also reduced by apabetalone (Additional file 3), while the abundance of *BRD2* or *BRD3* transcripts was unaltered (data not shown). A decrease in BRD4 levels could potentially reduce BET-dependent transcriptional regulation in monocytes.

**Fig. 3:**
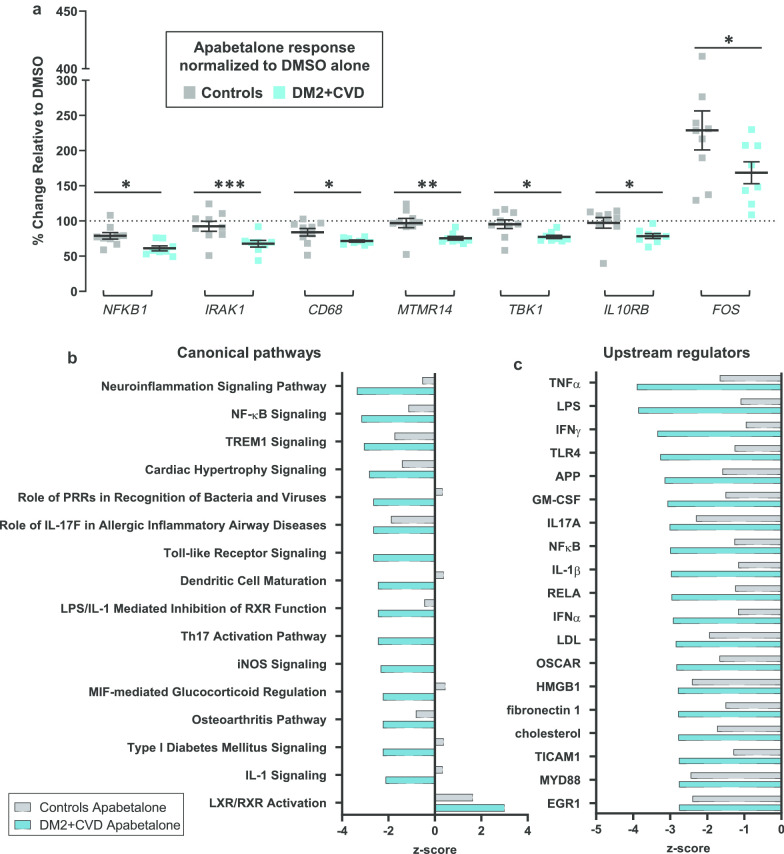
Enhanced downregulation of inflammatory mediators and pathways by apabetalone in ex vivo-treated DM2 + CVD monocytes as compared to controls. **a** Transcription of several genes is suppressed more robustly by apabetalone in monocytes from DM2 + CVD patients. mRNA expression levels are shown as % change following apabetalone treatment (25 μM) relative to the DMSO-treated baseline (100% dotted line). 2-Way repeated measures ANOVA with Bonferroni’s multiple comparisons test, *p < 0.05, **p < 0.01, ***p < 0.001. **b**, **c** Predicted effect of apabetalone on **b** IPA® canonical pathways and **c** IPA® upstream regulators. IPA® output was based on the input of gene expression changes of more than 20% with apabetalone treatment (versus DMSO, *p* < 0.05). IPA® z-scores compare changes in gene expression (“activating” or “inhibiting”) in the experimental dataset to changes predicted by the literature.* z* < − 2 predicts a downregulation within a gene set associated with a canonical pathway or a transcriptional regulator. *iNOS* inducible nitric oxide synthase, *RXR* retinoid X receptor, *MIF* macrophage migration inhibitory factor, *LXR* liver X receptor, *LPS* lipopolysaccharide, *APP* amyloid protein precursor, *EGR1* early growth response protein 1

Overall, treatment of DM2 + CVD monocytes with 25 μM apabetalone altered the abundance of 39 out of 109 transcripts detected by the NanoString Innate Immunity Panel (> 30% change with adjusted p < 0.05) (Table 2). 36 of 39 transcripts were downregulated, whereas 3 of them were upregulated by apabetalone treatment (Table 2). Ten gene transcripts were also sensitive to 5 μM apabetalone (marked by asterisks in Table 2). Gene transcription in control monocytes was similarly altered by apabetalone, but with several notable exceptions (Fig. 3a). Target genes associated with NF-κB pathway activation, including TANK-binding kinase 1 (*TBK1*) and IL-1 receptor-associated kinase 1 (*IRAK1*), as well as the NF-κB subunit 1 (*NFKB1*), were more efficiently suppressed by apabetalone in DM2 + CVD monocytes versus controls. Similarly, apabetalone differentially reduced gene expression of the phagocytic macrophage receptor *CD68* [41], of the IL-10 receptor subunit *IL10RB* implicated in pro- and anti-inflammatory homeostasis [42], and of *MTMR14* encoding a phosphoinositide phosphatase involved in metabolic dysregulation in obesity [43]. *FOS,* a transcription factor involved in myeloid differentiation [44], was induced by apabetalone treatment to a lesser extent in DM2 + CVD cells compared to control monocytes (Fig. 3a).

**Table 2:**
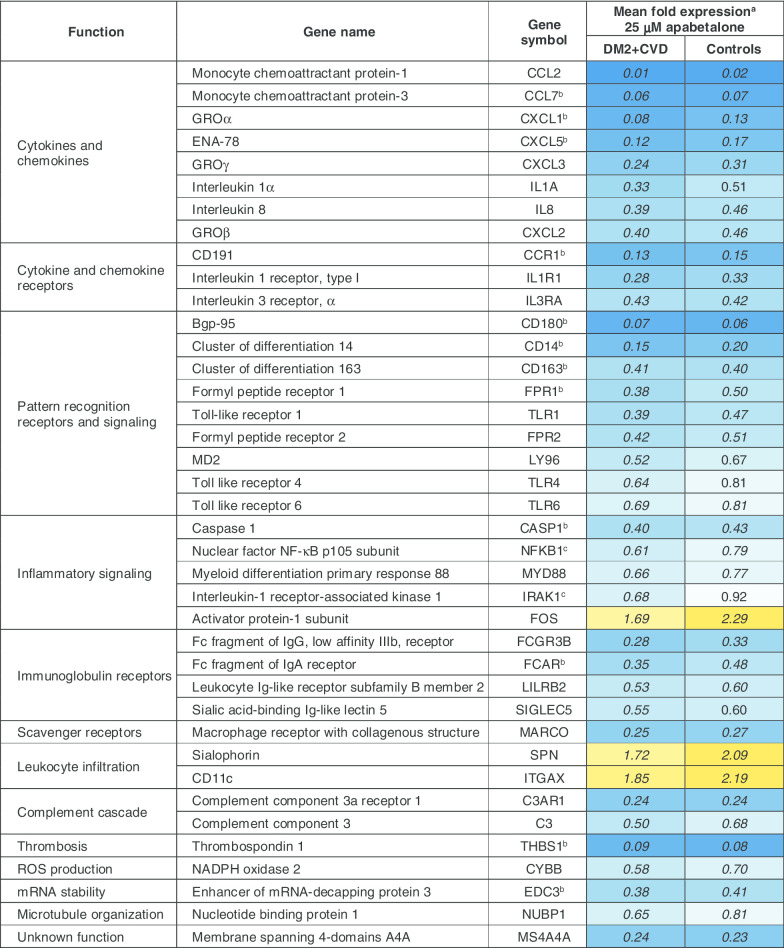
Apabetalone suppresses the expression of inflammatory genes in unstimulated monocytes obtained from DM2 + CVD patients and control subjects

^a^Gene expression is expressed as mean fold difference in response to 25 μM apabetalone treatment relative to vehicle (DMSO). ^b^Genes significantly downregulated in response to 5 μM apabetalone. ^c^Genes differentially sensitive to apabetalone treatment in DM2 + CVD versus control cells (also shown in Fig. 3a). Italics numbers indicate a fold change of > 30% with an adjusted *p* value < 0.05 (two-way repeated measures ANOVA)

Apabetalone target genes (> 20% change, *p* < 0.05 following a 4 h treatment) were further examined with Ingenuity® pathway analysis (IPA®) software to predict affected canonical pathways and upstream regulators (Fig. 3b, c). Apabetalone was predicted to downregulate key canonical pathways associated with monocyte inflammatory responses, such as the NF-κB signalling, the TLR signalling, the IL-1 signalling and the NLRP3 inflammasome pathway (Fig. 3b and Additional file 4). IPA® upstream regulator analysis predicted that apabetalone would suppress multiple transcriptional targets associated with cytokine signalling (TNFα, IFN, IL-17, IL-1, GM-CSF) and TLR signalling (LPS, TLR4, NF-κB, RELA, HMGB1, TICAM2, MYD88) (Fig. 3c and Additional file 5). Apabetalone had a greater inhibitory impact on these pathways and regulators in DM2 + CVD monocytes than in controls (as indicated by *z*-scores < −2 predicting a significant suppression of Upstream Regulator targets) (Fig. 3b, c). The data suggest that monocytes from diseased patients are activated via BET protein-dependent pathways and that these monocytes are more sensitive to BET inhibition by apabetalone than those of matched subjects without CVD or DM2.

### Apabetalone counters DM2 + CVD monocyte hyper-responsiveness to IFNγ

**Fig. 4:**
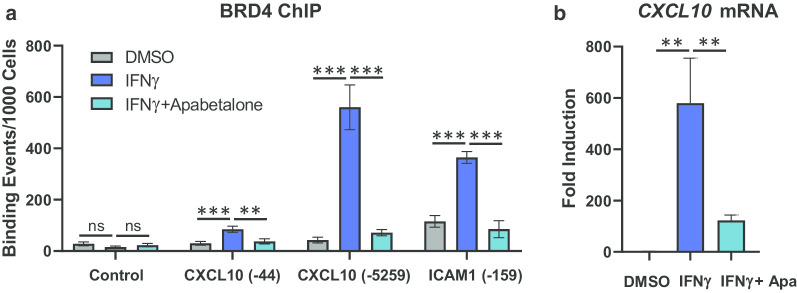
Apabetalone treatment decreased BRD4 occupancy at transcription regulatory elements of IFNγ-responsive genes. IFNγ stimulation (4 h) increases BRD4 occupancy on the *CXCL10* and *ICAM1* gene regulatory regions, but not in a BRD4 protein-lacking region (Control) as determined by chromatin immunoprecipitation (ChIP). Co-treatment with apabetalone (25 μM) reduces BRD4 association with gene regulatory regions. **b** Apabetalone (25 μM) also suppressed *CXCL10* mRNA transcript induction by IFNγ in these same samples. Samples were processed in triplicate. Data are presented as the mean ± S.D. Statistical significance was determined through ANOVA followed by Tukey’s Multiple Comparison Test, where **p < 0.01, ***p < 0.001 and ns, no significant difference

IFNγ is a pro-inflammatory cytokine produced by immune cells in the atherosclerotic plaque that initiates monocyte polarization into pro-inflammatory and tissue-destroying M1 macrophages [37]. BETi have previously been shown to regulate IFNα signalling in monocytes through inhibition of BRD4 association with gene expression regulatory elements [45]. To determine if apabetalone could impact BRD4 chromatin binding in response to IFNγ, we probed BRD4 occupancy of two IFNγ-sensitive genes, *CXCL10* and *ICAM1*, in THP-1 monocytes [46, 47]. As expected, cytokine stimulation (4 h) induced a significant enrichment of BRD4 on the *CXCL10* promoter (− 44 bp, 2.8-fold) and enhancer (− 5259 bp, 13-fold), but not in a BRD4-lacking control region (Fig. 4a). A similar induction was observed for the *ICAM1* gene promoter (− 159 bp, 3.2-fold). This BRD4 enrichment was countered by co-treatment with apabetalone (56%, 87% and 77% reduction, respectively), indicating that it was BD-dependent. Inhibition of BRD4 chromatin occupancy by apabetalone was consistent with the decrease in *CXCL10* gene transcript levels measured after treatment (Fig. 4b). The *ICAM1* mRNA transcript was not detectable 4 h post-induction (not shown).

**Fig. 5:**
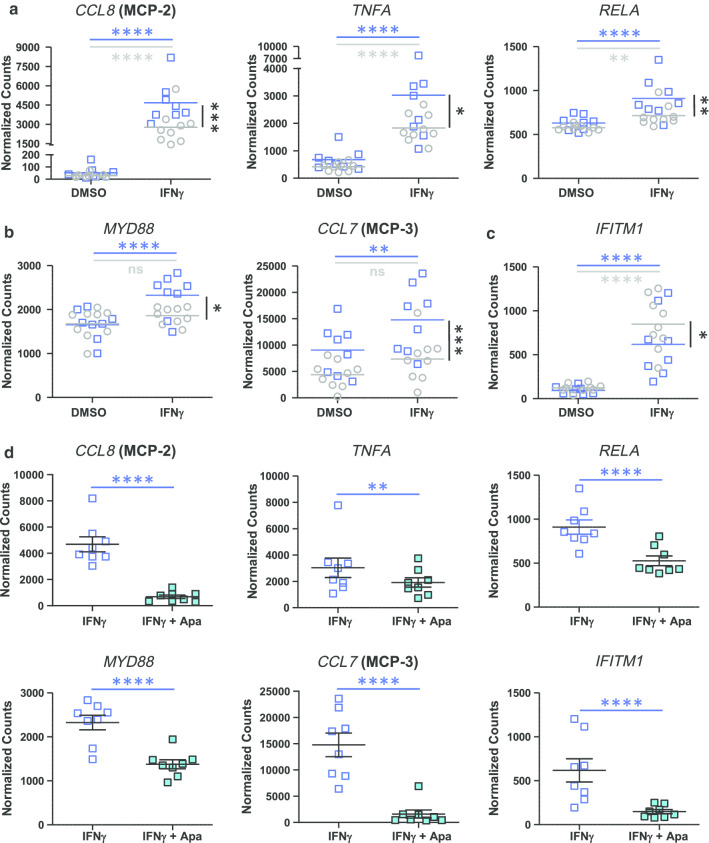
Genes hyper-sensitive to IFNγ stimulation are inhibited by 25 μM apabetalone (Apa) in DM2 + CVD monocytes. **a** Hyper-activation of the transcriptional response to IFNγ in monocytes obtained from DM2 + CVD patients (blue squares) compared to control cohort (grey circles). Gene expression changes are presented as means of endogenous control-normalized counts (Nanostring). **b** *MYD88* and *CCL7* are IFNγ inducible in DM2 + CVD monocytes only. **c** *IFITM2* is hyper-responsive in control monocytes. **d** Genes differentially responsive to IFNγ are suppressed by apabetalone. Endogenous control-normalized counts are shown (NanoString). Statistics: 2-Way repeated measures ANOVA followed by Tukey’s multiple comparisons test (within-group comparisons; blue bars and asterisks) or Bonferroni’s test (between-group comparison; black bars and asterisks); *p < 0.05; **p < 0.01; ***p < 0.001; ****p < 0.0001; *ns* non-significant. Individual patient data were shown as a mean ± SEM

To compare cell responses to IFNγ, DM2 + CVD and control monocytes were treated ex vivo with IFNγ for 4 h, followed by gene expression and protein secretion analysis. Significantly, IFNγ evoked a greater induction of pro-inflammatory genes *CCL8, TNF* and *RELA*, relative to baseline, in DM2 + CVD monocytes as compared to control monocytes (blue squares versus grey circles) (Fig. 5a, between-group statistical significance is indicated by black vertical bars and asterisks). *MYD88* and *CCL7* transcripts were increased by IFNγ only in DM2 + CVD monocytes; no significant change was detected in control monocytes, indicating differential sensitivity to the cytokine treatment between cohorts (Fig. 5b) (blue versus grey horizontal bars and asterisks). *IFITM1* transcripts increased in both monocyte populations with IFNγ stimulation, but were enhanced more in control monocytes (Fig. 5c). Overall, these data indicate that DM2 + CVD monocytes are hyperactivated by the pro-M1 stimulant IFNγ, leading to an enhanced expression of chemokines and genes within the NF-κB pathway.

**Fig. 6:**
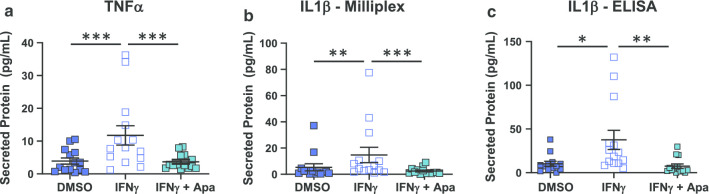
Apabetalone counters stimulated cytokine secretion in DM2 + CVD monocytes. **a**, **b** Secretion of proteins induced by IFNγ in DM2 + CVD monocytes is countered by 25 μM apabetalone (Apa) 24 h post-treatment (Milliplex®). **c** Secretion of IL-1b in DM2 + CVD monocytes was confirmed by ELISA. Statistics: 2-Way repeated measures ANOVA followed by Tukey’s multiple comparisons test; *p < 0.05; **p < 0.01; ***p < 0.001. Individual patient data were shown as a mean ± SEM

**Fig. 7:**
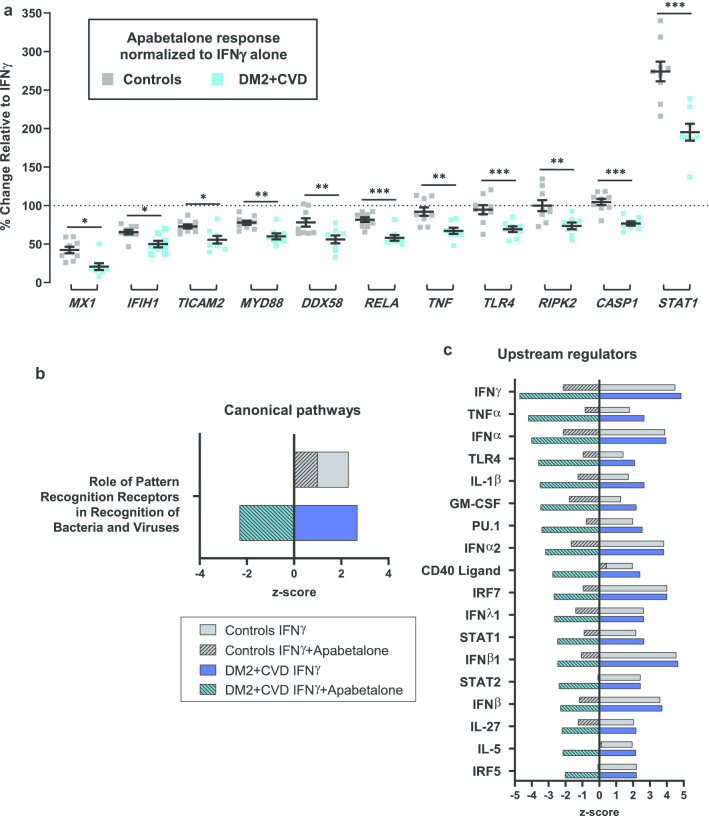
Differential effects of apabetalone treatment on IFNγ-sensitive inflammatory mediators and pathways in DM2 + CVD monocytes as compared to controls. **a** Apabetalone suppresses select IFNγ-induced genes more robustly in monocytes from DM2 + CVD patients as compared to controls. Apabetalone’s effect on gene expression in the presence of IFNγ was calculated relative to IFNγ only condition (100% dotted line). Statistical significance was determined using 2-way repeated measures ANOVA with Bonferroni’s multiple comparisons test, *p < 0.05, **p < 0.01, ***p < 0.001. **b**, **c** Predicted effect of IFNγ and IFNγ + apabetalone treatment on IPA®-curated canonical pathways and upstream regulators. IPA® output was based on the input of gene expression changes of more than 20% with apabetalone treatment (versus DMSO, *p* < 0.05). IPA® z-scores compare changes in gene expression (“activating” or “inhibiting”) in the experimental dataset to changes predicted by the literature. z < −2 predicts a downregulation and* z* > 2 predicts an upregulation within a gene set associated with a canonical pathway or a transcriptional regulator. *n/d* no predicted directional change, *IRF* interferon response factor; *PRRs* pattern recognition receptors, *RIG-I* retinoic acid-inducible gene I

Previous reports have indicated that IFNγ signalling is sensitive to BET inhibition [25, 45, 48, 49]. Ex vivo treatment with apabetalone suppressed the transcription of genes that responded differentially to IFNγ, namely *CCL8*, *TNF*, *RELA*, *MYD88*, *CCL7* and *IFITM1* (Fig. 5d and italics gene symbols in Table 3). The drug also countered IFNγ-stimulated secretion of the TNFα protein, consistent with gene expression data in DM2 + CVD monocytes (Fig. 6a). Secretion of IL-1β, as measured by Milliplex® and ELISA assays, also declined with apabetalone treatment (Fig. 6b, c, respectively), even though the *IL1B* transcription was not induced by IFNγ nor suppressed by apabetalone (data not shown). This may be due to apabetalone-mediated downregulation of the *CASP1* gene which is required for secretion of mature IL-1β (Tables 2, 3; see Discussion) [50]. While apabetalone reduced the transcription of numerous genes induced by IFNγ in both cohorts (Table 3), several IFNγ-induced genes were differentially suppressed in DM2 + CVD monocytes as compared to controls (Fig. 7a). In most cases, apabetalone suppressed inflammatory gene transcription more efficiently in DM2 + CVD monocytes (*MX1*, *IFIH1*, *TICAM2*, *MYD88*, *RELA*)*.* Transcripts of several genes (*DDX58*, *TNFA*, *TLR4*, *RIPK2* and *CASP1*) were significantly decreased by BETi treatment only in DM2 + CVD monocytes (Fig. 7a, Table 3). Uniquely, induction of IFNγ target gene STAT1 was differentially enhanced by apabetalone (Fig. 7a, Table 3). Overall, these data suggest that the transcriptional response induced by IFNγ has a greater BET-dependency and is more sensitive to apabetalone in DM2 + CVD monocytes than in control cells.

**Table 3:**
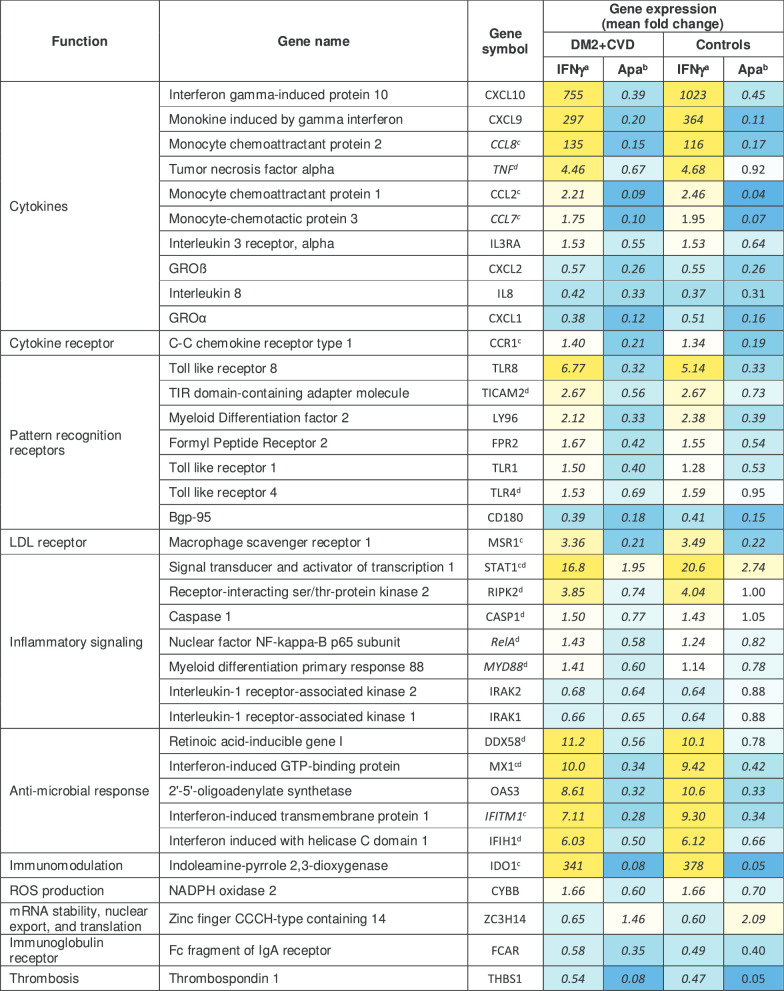
Ex vivo treatment with apabetalone counters IFNγ-mediated induction of gene expression in monocytes

^a^Gene induction in response to IFNγ is expressed as a mean fold change relative to vehicle (DMSO). ^b^Effect of 25 μM apabetalone in the presence of IFNγ is shown as a mean fold change in gene expression relative to IFNγ stimulation alone. ^c^Genes significantly downregulated in response to 5 μM apabetalone. Italics gene symbols indicate an enhanced response to IFNγ in DM2 + CVD monocytes (as compared to controls, also Fig. 5). ^d^Genes differentially sensitive to apabetalone treatment in DM2 + CVD versus control cells (also Fig. 7a). Italics numbers indicate fold change with an adjusted *p* value < 0.05 (2-way repeated measures ANOVA)

Functionally, the gene transcripts responsive to IFNγ and regulated by apabetalone can be categorized as cytokines, pattern recognition receptors, inflammatory signalling and anti-microbial response molecules (Table 3). Based on gene expression changes following IFNγ treatment of control and DM2 + CVD monocytes (> 20%, *p* < 0.05), IPA® predicted a significant upregulation of a canonical pathway linked to pattern recognition receptor (PRR) signalling (*p* < 0.0001, *z*-score > 2) (Fig. 7b and Additional file 6). Apabetalone treatment was predicted to significantly reverse the activation of the PRR signalling pathway only in DM2 + CVD monocytes (*z*-score < − 2) as no significant directional effect of apabetalone was predicted in control monocytes (2 > *z*-score > − 2) (Fig. 7b). The IPA® upstream regulator analysis showed that IFNγ target genes play a role downstream of key cytokines and transcription factors involved in macrophage differentiation and activation (Fig. 7c and Additional file 7). While apabetalone also had a significant inhibitory effect on the IFNγ-dependent inflammatory pathways in control monocytes (Fig. 7c; grey hatched bars), the predicted suppression of gene signatures was more pronounced in DM2 + CVD monocytes (Fig. 7c; teal hatched bars). Taken together, IFNγ-mediated gene transcription was countered by BET inhibition more efficiently in DM2 + CVD monocytes as compared to controls. Overall, our findings suggest that the transcriptional responsiveness of IFNγ-regulated genes is more dependent on BET proteins in the diseased state.

## Discussion

In this study, we compared the pro-inflammatory activity of monocytes derived from DM2 + CVD patients on standard of care therapy versus matched control subjects. Monocytes from DM2 + CVD patients exhibited a hyper-inflammatory state characterized by increased gene expression of pro-inflammatory cytokines IL-8*,* IL-1α and IL-1β and of the IgA receptor FCAR (also known as CD89). At the protein level, we observed enhanced surface expression of TLR2 in intermediate (CD14^++^CD16^+^) and non-classical (CD14^+^CD16^+^) DM2 + CVD monocytes and increased secretion of chemoattractants IL-8 and GROα (*CXCL1*) (Fig. 1). These changes in gene and protein abundance suggest that the monocytes circulating in DM2 + CVD patients adopt a pro-inflammatory phenotype despite standard of care therapy including statins.

Enhanced TLR2 expression had previously been reported on monocytes from DM2 and metabolic syndrome patients [38–40]. TLR2 is a pattern recognition receptor that recognizes molecular danger signals that are linked to microbial infection or sterile tissue injury. It is also crucial in the initiation and progression of atherosclerosis, since it stimulates foam cell formation [51]. Statins are reported to decrease monocyte TLR expression in DM patients [52–54]. However, statin therapy did not normalize monocyte TLR2 expression in our DM2 + CVD cohort. TLR2 activation induces the expression of cytokines, chemokines and cell adhesion molecules by immune cells, promoting na pro-inflammatory phenotype [55]. Similarly, FCAR activation by immunoglobulin A enhances production of pro-inflammatory cytokines, prostaglandins and leukotrienes [56–58]. Enhanced TLR and/or FCAR surface expression may underlie the observed hyperactive transcription of *IL1A*, *IL1B* and *CXCL8* as well as enhanced secretion of IL-8 and GROα in DM2 + CVD monocytes observed in this study. Elevated levels of these cytokines have previously been described in DM2 and CVD patients [59–61]. These pro-inflammatory mediators are highly pro-atherogenic as they promote differentiation to a M1-like macrophage phenotype, enhanced vascular wall recruitment and increased endothelial transmigration [62–66]. Thus, their overproduction likely contributes to the pathogenesis of atherosclerosis in DM2 + CVD patients on standard of care therapy.

A number of genes were downregulated in monocytes isolated from DM2 + CVD patients (relative to controls), including the mRNA splicing complex encoding gene *SF3A3*, the scavenger receptor *MARCO* and the plasma membrane tetraspan gene *MS4A4*. The latter two genes are expressed on monocytes with anti-inflammatory M2-like characteristics [67, 68], whereas the *SF3A3* gene negatively regulates pro-inflammatory TLR signalling [69]. Thus, downregulation of these genes may indicate reprogramming towards a pro-inflammatory phenotype in patients. Upon IFNγ stimulation, monocytes acquire a M1-like phenotype characterized by enhanced cytokine production, phagocytosis and intracellular killing of microbial pathogens [70]. We show that monocytes isolated from DM2 + CVD patients are more responsive to IFNγ ex vivo treatment, with enhanced expression of genes that encode the NF-κB inflammatory pathway components (*TNF*, *MYD88* and *RELA*) and chemokines (*CCL7* and *CCL8*). Our observations are consistent with previous studies reporting that monocytes from CVD patients (53% of whom also had DM2) overproduce cytokines in response to treatment with IFNγ combined with lipopolysaccharide [8]. Overall, the observed gene expression and secretory profile of DM2 + CVD monocytes indicate a potential phenotypic shift from the M2-like state towards the pro-inflammatory M1-like state.

This persistent hyper-responsive phenotype is likely linked to changes to the epigenetic landscape characteristic of cardiometabolic disease [14, 19]. In human monocytes exposed to hyperglycemic conditions ex vivo, there is a decrease in transcription inhibiting histone methylation marks and an increase in open chromatin marks, potentially enhancing transcription factor accessibility to DNA [17, 71, 72]. Congruently, in monocytes from diabetic patients, transcription-activating acetylation marks are more abundant on the promoters of pro-inflammatory genes encoding TNFα and cyclooxygenase-2 (*PTGS*) [71, 72]. Transcription-activating acetylation of histone 3 lysine 27 (H3K27) and methylation of H3K4 is also increased in immune and vascular smooth muscle cells isolated from atherosclerotic plaques in human carotids [73]. BET proteins “read” histone acetylation patterns including H3K27, thereby activating aberrant gene expression in diseased or stimulus-activated cells [23, 28, 30]. However, it is unclear if excessive BET activity contributes to pro-inflammatory monocyte response in DM2 + CVD patients.

Here, we demonstrate that the BET inhibitor apabetalone disrupts pro-inflammatory gene expression in hyperactive DM2 + CVD monocytes. Indeed, in unstimulated conditions, apabetalone suppresses numerous genes more efficiently in DM2 + CVD monocytes, including those associated with cytokine, TLR and NF-κB signalling. Of note, even in baseline conditions, apabetalone significantly reduces the IFNγ transcriptional signature in DM2 + CVD monocytes (but not in control cells), suggesting that IFNγ target genes are hyperactive in diabetic monocytes. Our data agrees with previously published observations, showing that IFNγ target genes can be induced by high glucose in ex vivo cultured human monocytes [17]. Further, upon ex vivo IFNγ stimulation, the TLR pathway and the cytokine transcriptional networks were predicted to be downregulated by apabetalone, especially in DM2 + CVD monocytes (Fig. 7). This differential response to apabetalone treatment suggests that BET proteins drive the disease-associated IFNγ signature in DM2 + CVD patients’ monocytes. Interestingly, monocytes from systemic sclerosis patients also display BETi-sensitive IFN signatures, similarly to the DM2 + CVD monocytes studied here [74]. We have shown that apabetalone can dislodge BRD4 from chromatin during IFNγ-mediated induction of model genes *CXCL10* and *ICAM1* in an in vitro monocyte model, providing insight into the drug’s mechanism of action. However, further epigenetic studies will be required to explain the differential sensitivity to BET inhibition between non-diseased and diseased cells.

Macrophages present in the atherosclerotic plaque are largely derived from circulating monocytes infiltrating blood vessels’ walls [7, 75]. In mice, monocytes have been shown to enter into the plaque more readily in the context of diabetes and hypercholesterolemia [76–78]. In non-stimulated conditions, apabetalone suppressed protein secretion of key monocyte chemoattractants MCP-1, MCP-3, GRO-α and IL-8 (87%, 79%, 55% and 32% reduction in DM2 + CVD monocytes, respectively). Apabetalone treatment also potently downregulated the transcription of genes differentially induced by IFNγ in DM2 + CVD monocytes, including the pro-inflammatory cytokine *TNF* gene (33% reduction), and monocyte chemokine genes *CCL7* and *CCL8* (90% and 85% reduction, respectively). Additional chemokines that promote chemotaxis and tissue extravasation, *CCL2* [62, 64]*, CXCL9* [79] and *CXCL10* [79], were induced by IFNγ in both monocyte populations and were strongly suppressed by apabetalone (91%, 80% and 61% reduction, respectively) (Table 3). BETi treatment is thus predicted to reduce the migratory phenotype of DM2 + CVD monocytes, potentially preventing atherosclerotic plaque infiltration by activated monocytes.

Upon stimulation with cytokines and PRR agonists, monocytes and macrophages activate the inflammasome pathway, which is responsible for the production of the mature secreted form of IL-1β, a major pro-atherosclerotic cytokine [50]. IFNγ contributes to high *IL1B* gene expression only when combined with secondary stimuli [80]. Consistent with published data [80], IFNγ did not change the *IL1B* transcript abundance as a single agent (data not shown), but it enhanced the IL-1β protein secretion by 2.7-fold in DM2 + CVD monocytes (Fig. 6). This induction was countered by apabetalone treatment (84% reduction). IL-1β is synthesized by monocytes in its inactive form (pro-IL-1β), which is then converted into its active mature form by the NLRP3 inflammasome-associated caspase 1 protease (encoded by *CASP1* gene) [50]. Apabetalone (25 μM) downregulated *CASP1* gene transcription in DM2 + CVD monocytes in non-stimulated (Table 2) and stimulated (Table 3) conditions, potentially explaining the observed decrease in IL-1β. The severity of atherosclerosis correlates with inflammasome activity and NLRP3/caspase 1-mediated generation of IL-1β and IL-1α in human atherosclerotic plaque [81]. Interestingly, the anti-IL-1β monoclonal antibody canakinumab reduced the risk of MACE for CVD patients by 15% in 3.7 years, independently of lipid lowering [82]. This proof-of-concept study demonstrated that countering inflammasome-mediated inflammation significantly reduces residual CVD risk, promoting the development of new anti-inflammatory therapeutic agents.

## Conclusions

We have demonstrated that monocytes isolated from DM2 + CVD patients exhibit a hyper-inflammatory phenotype at baseline and a hyper-responsiveness to inflammatory stimuli ex vivo. This activated monocyte state may contribute to the initiation and progression of atherosclerosis and increased cardiovascular risk in these patients. Ex vivo BET-inhibition by apabetalone reduces this enhanced pro-inflammatory phenotype, providing a rationale for further evaluation of BET inhibitors as therapeutic agents for high risk DM2 + CVD patients. Previously, a post hoc analysis of pooled phase 2 trials of CVD patients indicated that apabetalone reduced MACE (5.9% in the treatment group compared to 10.4% in the placebo group; *p* = 0.02) [83]. In a subgroup analysis of patients with DM2, apabetalone further reduced the MACE hazard ratio to 0.38 (95% CI 0.15–0.99; *p* = 0.04) [83]. In the recently completed phase 3 cardiovascular outcomes trial (BETonMACE), 2425 patients with DM2 and recent acute coronary syndrome were treated with apabetalone or placebo, and followed for 26 months [84, 85]. Although the primary endpoint of the study, a reduction in time to first occurrence of MACE defined as CV death, non-fatal myocardial infarction or stroke, did not achieve statistical significance, apabetalone did demonstrate benefits on hospitalization for heart failure [85]. As monocyte activation and infiltration contributes to heart failure, apabetalone treatment may counter the detrimental immune inflammatory response in patients with post-acute coronary syndrome and DM2.

## Materials and methods

### Study design

This observational mono-centre cohort study enrolled subjects diagnosed with DM2 and high CVD risk (myocardial infarction, percutaneous coronary intervention, coronary artery bypass graft, peripheral arterial disease, episode of unstable angina, transient ischemic event, cerebrovascular accident or peripheral arterial disease, but event-free in past 3 months) and age and gender matched volunteers (Table 1). Exclusion criteria consisted of a history of chronic kidney disease stage 3b-5, malignant diseases or any clinically significant medical condition within the past 2 years that could interfere with the conduct of the study in the opinion of the investigator, treatment with immunosuppressants within the 3 months prior to visit 1 and evident drug or alcohol use. The study protocol was approved by the medical ethical committee of the Amsterdam Medical Centre in Amsterdam, the Netherlands. Each subject provided written informed consent.

### Biochemical measurements

Blood was collected while patients were in a fasting state. Plasma total cholesterol, high-density lipoprotein (HDL) cholesterol, triglycerides and lipoprotein(a) levels were analysed using commercially available methods. Low-density lipoprotein cholesterol levels were calculated using the Friedewald equation.

### PBMC isolation and monocyte culture

Peripheral blood mononuclear cells (PBMCs) were isolated from DM2 + CVD subjects and healthy controls by Ficoll density gradient centrifugations (Axis-Shield) as described in detail previously [86]. In brief, after washing PBMCs, CD14^+^ monocytes were isolated using human CD14 magnetic beads and MACS® cell separation columns according to manufacturer’s instructions (Miltenyi Biotec, Leiden, The Netherlands). Then, these CD14^+^ monocytes were ex vivo treated with IFNγ and/or apabetalone, and phenotyped for gene expression (NanoString) and protein secretion (Millipore Milliplex® Human Cytokine/Chemokine Array).

### Flow cytometry

Whole blood was collected from DM2 + CVD patients and matched controls in EDTA tubes. After lysis of red blood cells with red blood cell lysis buffer 10× (eBioscience), white blood cells were stained with antibodies for cell surface markers CCR2, CD11c, CD36, CD29, CCR5, CD33, CD32, TLR2, CD11b,CCR7, CD163, TLR4, HLA-DR, CD14, CD16, IVIG. Fluorescence was measured using a FACS CANTO II (BD) and analysed with FlowJo software version V10.6. Monocyte area was gated based on forward and side scatter, CD14^+^ and/or CD16^+^ and HLA-DR. Monocytes were classified as classical (CD14^++^CD16^−^), intermediate (CD14^++^CD16^+^) or non-classical (CD14^+^CD16^+^). The expression of cell surface markers was calculated as the delta geometric mean (∆GM). ∆GM = GM surface staining—GM unstained control. Statistical differences in cell surface marker abundance between DM2 + CVD patient monocytes and controls were determined using a Student’s *t*-test.

### Multiplexed gene expression analysis via nanostring

CD14^+^ monocytes from 8 DM2 + CVD patients and 9 control subjects were pre-incubated with DMSO (0.025%) or apabetalone (5 or 25 μM) for 1 h, followed by IFNγ (25 U/ml) co-stimulation for 4 h. Non-stimulated samples received DMSO or apabetalone for 4 h. Monocytes were lysed with TriPure (Roche, Basel, Switzerland), and total RNA was isolated and analysed using the nCounter® Vantage 3D™ Innate Immunity Panel (NanoString) (University of Alberta) for multiplexed single molecule counting of 180 human gene transcripts. Data were analysed using nSolver™ and Ingenuity® Pathway Analysis (IPA®). IPA® z-scores compare the observed differential regulation of a gene (“activating” or “inhibiting”) in the dataset to changes predicted by the literature. *z* < − 2 predicts a downregulation, and *z* > 2 predicts an upregulation within a gene set associated with a canonical pathway or a transcriptional regulator. *p* value < 0.01 indicates a statistically significant overlap between the dataset genes and the curated gene sets (Fisher’s exact test).

### Real-time PCR

To assess BRD2, BRD3 and BRD4 expression, total RNA extracted from monocytes was reverse transcribed with High-Capacity cDNA RT Kit (Thermofisher Scientific) and amplified using TaqMan™ Gene Expression Master Mix and TaqMan Real-Time PCR assays (Applied Biosystems). Gene expression was normalized to the endogenous control cyclophilin A (duplex reaction). Data were acquired using the ViiA-7 rtPCR System (Applied Biosystems).

### Multianalyte immunoprofiling

In unstimulated conditions, monocytes were treated with DMSO (0.025%, vehicle control) or with apabetalone (25 μM) for 24 h. In stimulated conditions, monocytes were incubated with DMSO, IFNγ (25 U/ml) + DMSO or IFNγ + apabetalone (25 μM) for 24 h. Supernatants were collected from triplicate treatments, pooled and analysed using the Millipore Milliplex® Human Cytokine Array / Chemokine Array 42-Plex with IL-18 (HD42) (Eve Technologies, Calgary, AB) to quantify EGF, Eotaxin-1, FGF-2, Flt-3L, Fractalkine, G-CSF, GM-CSF, GROα, IFNα2, IFNγ, IL-1α, IL-1β, IL-1RA, IL-2, IL-3, IL-4, IL-5, IL-6, IL-7, IL-8, IL-9, IL-10, IL-12 (p40), IL-12 (p70), IL-13, IL-15, IL-17A, IL-18, IP-10, MCP-1, MCP-3, MDC, MIP-1α, MIP-1β, PDGF-AA, PDGF-AB/BB, RANTES, sCD40L, TGFα, TNFα, TNFβ and VEGF-A.

### ELISA

Cytokine levels were measured in samples used for multianalyte immunoprofiling by commercially available enzyme-linked immunosorbent assay kits for MCP-1, IL-8 and IL-1β according to the manufacturer’s instructions (Invitrogen). High binding half area 96 well plates were used for these assays.

### Chromatin immunoprecipitation

To assess BET protein chromatin occupancy, THP-1 cells were pre-treated with BET inhibitors for 1 h before addition of IFNγ (25 U/ml) for a 4 h co-incubation period. After cross-linking cells with formaldehyde, Active Motif Inc. (Carlsbad, CA) performed chromatin isolation and immunoprecipitation with BRD4 antibodies (Bethyl). Samples were processed in triplicate. Statistical significance was determined through 2-way ANOVA followed by Tukey’s Multiple Comparison Test.

### Statistical analysis

Differences in gene expression were determined to be statistically significant by comparing endogenous control-normalized raw counts (calculated by nSolver™) using a 2-way repeated measures ANOVA test followed by Tukey’s multiple comparisons correction for within-group comparisons, or Bonferroni’s test for between-group comparisons (PRISM 8). Statistically significant differences in protein secretion were determined by comparing absolute concentrations (obtained by Milliplex® immunoprofiling). Percent change in gene expression was calculated versus each subject’s DMSO-treated sample or IFNγ-treated sample and compared between cohorts with 2-way ANOVA followed by Tukey’s test. Results are presented as mean or median values ± SEM. *p *value ≤ 0.05 was considered statistically significant.

## Supplementary information

**Additional file 1–3. Additional file 1.** Analysis of monocyte subpopulations in control (CTL) and DM2+CVD patients. Monocytes were classified as classical (CD14++CD16−), intermediate (CD14++CD16+) or non-classical (CD14+CD16+). Fluorescence was measured using a FACS CANTO II (BD) and analysed with FlowJo software. **Additional file 2.** Monocytes were stimulated ex vivo with IFNγ (1.5, 3.12, 6.25, 12.5 or 25 U/ml), apabetalone (1.5, 3.12, 6.25, 12.5 or 25 μM), or a combination of both stimuli for 24h. Subsequently, cytotoxicity was determined by measuring the enzyme lactate dehydrogenase (LDH) in the supernatant using the CytoTox 96® non-radioactive cytotoxicity assay (Promega). There was no difference between ‘unstimulated’ and ‘stimulated’ conditions, except for the positive control where *p* < 0.0001. Statistics: One-way ANOVA with Dunnett’s multiple comparisons test. **Additional file 3.** BRD4 mRNA expression is reduced by ex vivo treatment with apabetalone in DM2+CVD monocytes (4h ex vivo treatment, 25 μM). BRD4 expression was measured by real-time PCR and normalized to cyclophilin A (endogenous control). Statistics: Unpaired Student’s t-test, ****, *p * < 0.0001.

**Additional file 4–7. Additional file 4.** Apabetalone’s gene targets in IPA® canonical pathways gene sets: Unstimulated control and DM2+CVD monocytes treated with apabetalone ex vivo. **Additional file 5**. Apabetalone’s gene targets that converge on IPA® upstream regulators: unstimulated monocytes treated with apabetalone ex vivo. **Additional file 6.** Apabetalone’s gene targets within IPA® canonical pathways: IFNγ stimulated monocytes treated with apabetalone ex vivo. **Additional file 7.** Apabetalone’s gene targets in IPA® upstream regulators gene sets: IFNγ stimulated control and DM2+CVD patient monocytes treated with apabetalone ex vivo.

## Data Availability

All data generated or analysed during this study are included in this published article and its supplementary information files.

## Abbreviations

BD: Bromodomain
BET: Bromodomain and extraterminal
BETi: BET inhibitor
CASP1: NLRP3 inflammasome-associated caspase 1 protease
CVD: Cardiovascular disease
DM2: Type 2 diabetes mellitus
FCAR: Fc region of IgA
GM-CSF: Granulocyte-macrophage colony stimulating factor
GRO: Growth-regulated oncogene
HDL: High-density lipoprotein
IFNγ: Interferon gamma
IL: Interleukin
IL-1RA: Interleukin 1 receptor antagonist IL-1RA
IPA®: Ingenuity® Pathway Analysis
LDL: Low-density lipoprotein
MACE: Major acute cardiac event
MARCO: Macrophage receptor with collagenous structure
MCP: Monocyte chemoattractant protein
MS4A4A: Membrane-spanning 4-domains subfamily A member 4A
NF-κB: Nuclear factor κ-light-chain-enhancer of activated B cells
PBMC: Peripheral blood mononuclear cells
PRR: Pattern recognition receptor
ROS: Reactive oxygen species
TLR: Toll-like receptor
TNF: Tumor necrosis factor
SF3A3: Splicing factor 3A subunit 3
