# Supplementary material for: BET protein inhibitor apabetalone (RVX-208) suppresses pro-inflammatory hyper-activation of monocytes from patients with cardiovascular disease and type 2 diabetes
Source: Clin Epigenetics. 2020 Nov 11;12:166. doi: 10.1186/s13148-020-00943-0 (PMC7657365; doi:10.1186/s13148-020-00943-0)
Supplement: Supplementary file 1 — Additional file 1–3. Additional file 1. Analysis of monocyte subpopulations in control (CTL) and DM2+CVD patients. Monocytes were classified as classical (CD14++CD16−), intermediate (CD14++CD16+) or non-classical (CD14+CD16+). Fluorescence was measured using a FACS CANTO II (BD) and analysed with FlowJo software. Additional file 2. Monocytes were stimulated ex vivo with IFNγ (1.5, 3.12, 6.25, 12.5 or 25 U/ml), apabetalone (1.5, 3.12, 6.25, 12.5 or 25 μM), or a combination of both stimuli for 24h. Subsequently, cytotoxicity was determined by measuring the enzyme lactate dehydrogenase (LDH) in the supernatant using the CytoTox 96® non-radioactive cytotoxicity assay (Promega). There was no difference between ‘unstimulated’ and ‘stimulated’ conditions, except for the positive control where p < 0.0001. Statistics: One-way ANOVA with Dunnett’s multiple comparisons test. Additional file 3. BRD4 mRNA expression is reduced by ex vivo treatment with apabetalone in DM2+CVD monocytes (4h ex vivo treatment, 25 μM). BRD4 expression was measured by real-time PCR and normalized to cyclophilin A (endogenous control). Statistics: Unpaired Student’s t-test, ****, p < 0.0001. [file 13148_2020_943_MOESM1_ESM.pptx]

## Slide 1
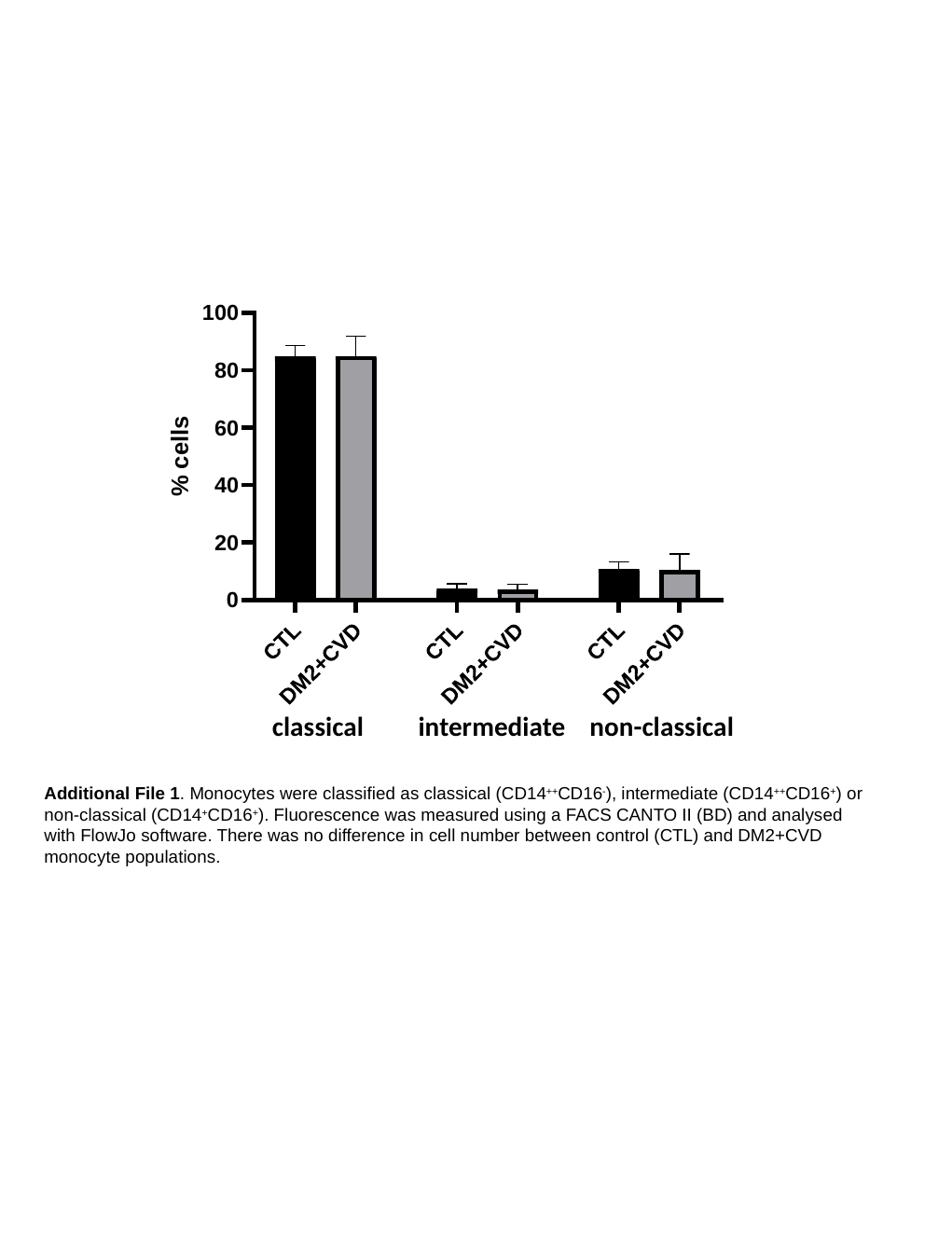

classical intermediate non-classical
Additional File 1. Monocytes were classified as classical (CD14++CD16-), intermediate (CD14++CD16+) or non-classical (CD14+CD16+). Fluorescence was measured using a FACS CANTO II (BD) and analysed with FlowJo software. There was no difference in cell number between control (CTL) and DM2+CVD monocyte populations.

## Slide 2
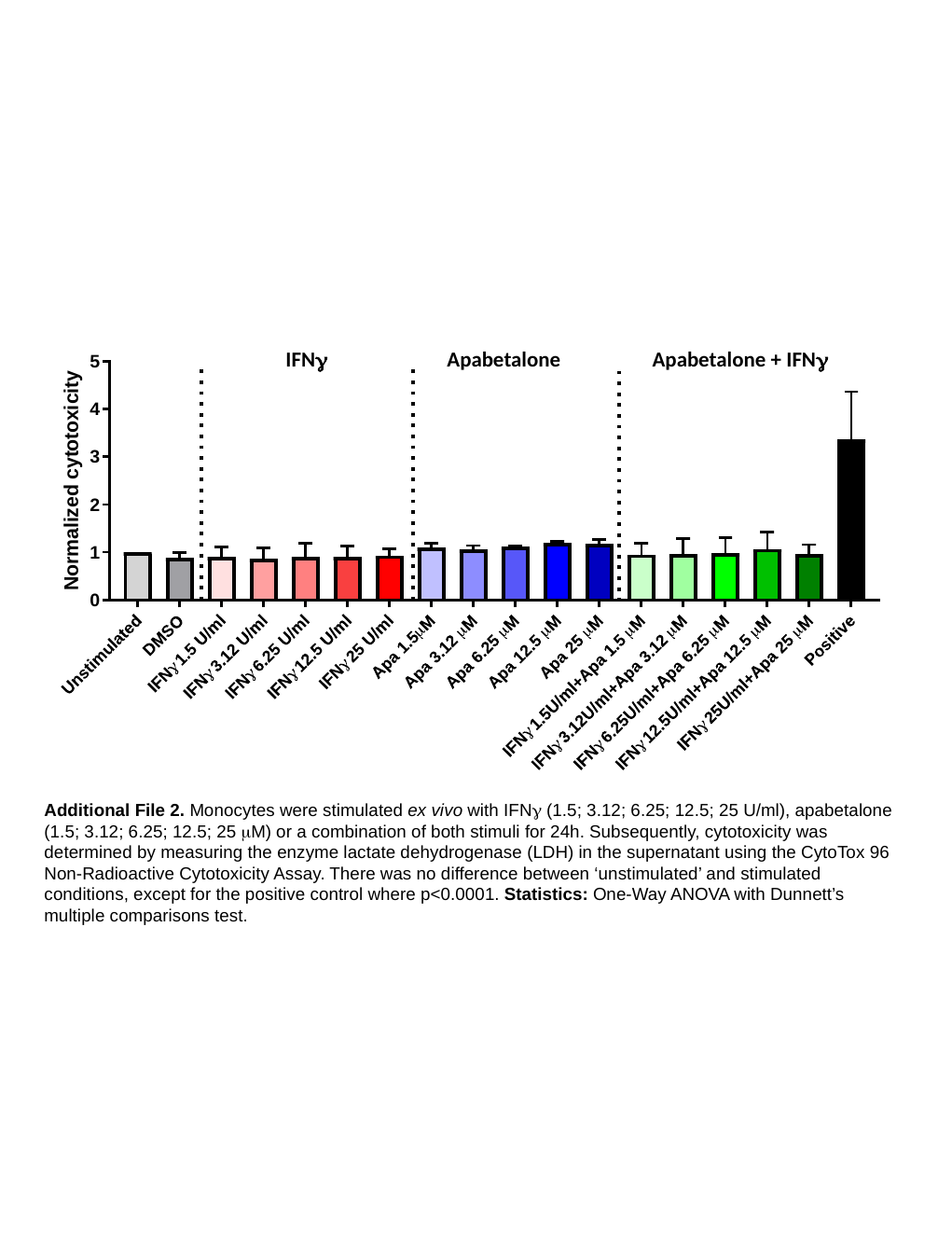

IFNg
Apabetalone
Apabetalone + IFNg
Additional File 2. Monocytes were stimulated ex vivo with IFNg (1.5; 3.12; 6.25; 12.5; 25 U/ml), apabetalone (1.5; 3.12; 6.25; 12.5; 25 mM) or a combination of both stimuli for 24h. Subsequently, cytotoxicity was determined by measuring the enzyme lactate dehydrogenase (LDH) in the supernatant using the CytoTox 96 Non-Radioactive Cytotoxicity Assay. There was no difference between ‘unstimulated’ and stimulated conditions, except for the positive control where p<0.0001. Statistics: One-Way ANOVA with Dunnett’s multiple comparisons test.

## Slide 3
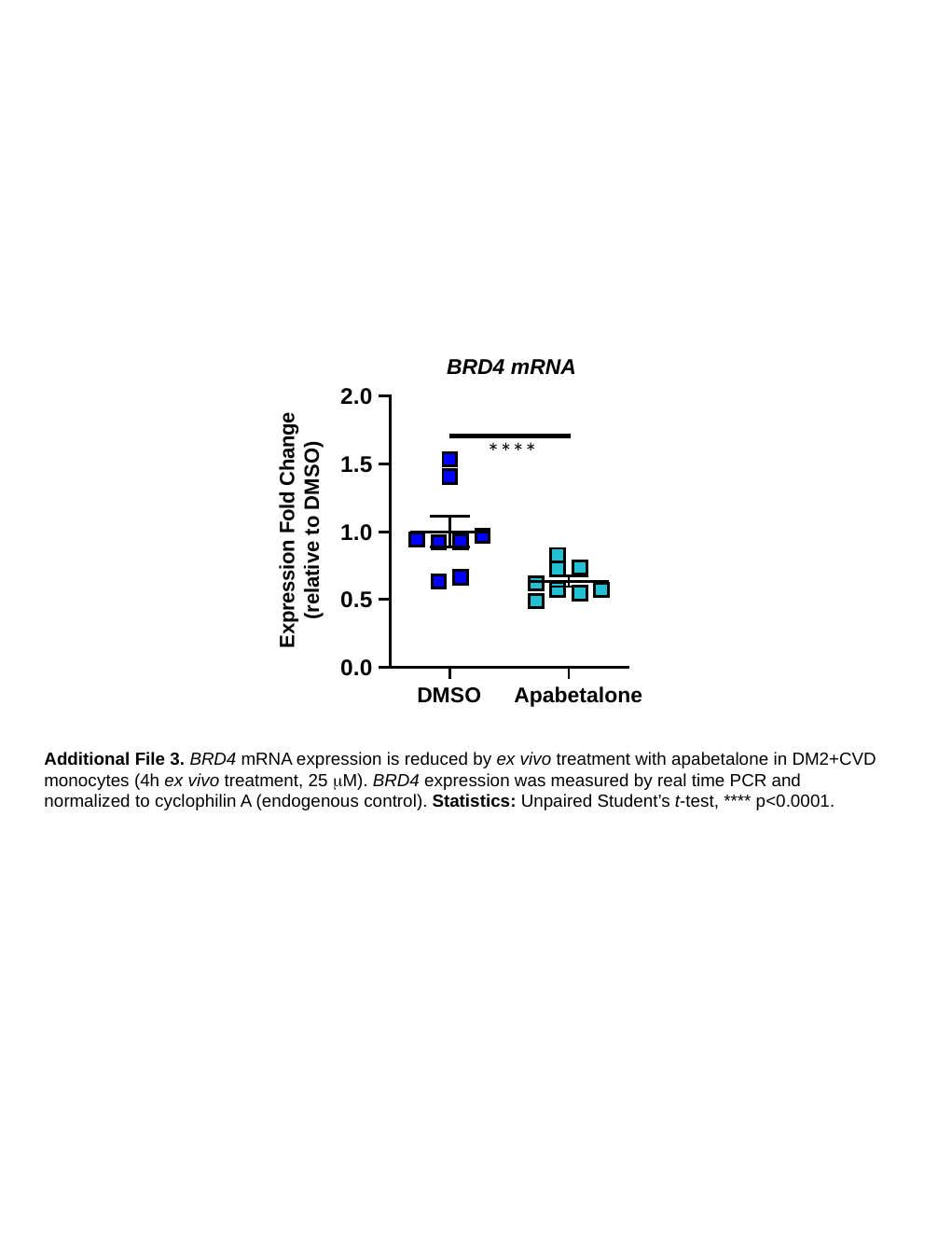

BRD4 mRNA
****
DMSO
Apabetalone
Additional File 3. BRD4 mRNA expression is reduced by ex vivo treatment with apabetalone in DM2+CVD monocytes (4h ex vivo treatment, 25 mM). BRD4 expression was measured by real time PCR and normalized to cyclophilin A (endogenous control). Statistics: Unpaired Student’s t-test, **** p<0.0001.
