# Supplementary material for: BET protein inhibitor apabetalone (RVX-208) suppresses pro-inflammatory hyper-activation of monocytes from patients with cardiovascular disease and type 2 diabetes
Source: Clin Epigenetics. 2020 Nov 11;12:166. doi: 10.1186/s13148-020-00943-0 (PMC7657365; doi:10.1186/s13148-020-00943-0)
Supplement: Supplementary file 2 — Additional file 4–7. Additional file 4. Apabetalone’s gene targets in IPA® canonical pathways gene sets: Unstimulated control and DM2+CVD monocytes treated with apabetalone ex vivo. Additional file 5. Apabetalone’s gene targets that converge on IPA® upstream regulators: unstimulated monocytes treated with apabetalone ex vivo. Additional file 6. Apabetalone’s gene targets within IPA® canonical pathways: IFNγ stimulated monocytes treated with apabetalone ex vivo. Additional file 7. Apabetalone’s gene targets in IPA® upstream regulators gene sets: IFNγ stimulated control and DM2+CVD patient monocytes treated with apabetalone ex vivo. [file 13148_2020_943_MOESM2_ESM.docx]

| **IPA® Canonical Pathways** | **z-score***  **CTL**  **Apa vs. DMSO** | **p-value‡** | **z-score***  **DM2+CVD Apa vs. DMSO** | **p-value‡** | **Apabetalone Targets in**  **DM2+CVD Monocytes#** |
| --- | --- | --- | --- | --- | --- |
| Neuroinflammation Signaling Pathway | -0.54 | 3.29E-13 | -3.36 | 1.70E-15 | CASP1, CCL2, CXCL8, CYBB, FOS, IL1R1, IRAK1, IRAK2, MYD88, NFKB1, RELA, TBK1, TLR1, TLR4, TLR6 |
| NF-κB Signaling | -1.13 | 1.38E-06 | -3.16 | 4.01E-11 | IL1A, IL1R1, RAK1, MYD88, NFKB1, RELA, TBK1, TLR1, TLR4, TLR6 |
| TREM1 Signaling | -1.73 | 3.11E-17 | -3.05 | 3.26E-20 | CASP1, CCL2, CCL7, CXCL3, CXCL8, IRAK1, ITGAX, MYD88, NFKB1, RELA, TLR1, TLR4, TLR6 |
| Cardiac Hypertrophy Signaling (Enhanced) | -1.41 | 6.98E-05 | -2.83 | 3.00E-05 | CXCL8, CYBB, IL10RB, IL1A, IL1R1, IL3RA, NFKB1, RELA |
| Role of PRRs in Recognition of Bacteria and Viruses | 0.33 | 1.85E-12 | -2.65 | 1.09E-11 | C3AR1, CASP1, CXCL8, IL1A, MYD88, NFKB1,RELA, TLR1, TLR4, TLR6 |
| Role of IL-17F in Allergic Inflammatory Airway Diseases | -1.89 | 1.90E-10 | -2.65 | 5.54E-11 | CCL2, CCL7, CXCL1, CXCL5, CXCL8, NFKB1, RELA |
| Toll-like Receptor Signaling | 0.00 | 9.00E-14 | -2.65 | 2.74E-18 | CD14, FOS, IL1A, IRAK1, IRAK2, LY96, MYD88, NFKB1, RELA, TLR1, TLR4, TLR6 |
| Dendritic Cell Maturation | 0.38 | 1.55E-06 | -2.45 | 1.15E-05 | FCGR3A/FCGR3B, IL1A, MYD88, NFKB1, RELA, TLR4 |
| LPS/IL-1 Mediated Inhibition of RXR Function | -0.45 | 6.31E-04 | -2.45 | 2.45E-06 | CD14, IL1A, IL1R1, IRAK1, LY96, MYD88, TLR4 |
| Th17 Activation Pathway |  | 3.41E-03 | -2.45 | 2.58E-07 | IL1R1, IRAK1, IRAK2, MYD88, NFKB1, RELA |
| iNOS Signaling | 0.00 | 1.72E-08 | -2.33 | 1.87E-14 | CD14, FOS, IRAK1, IRAK2, LY96, MYD88, NFKB1, RELA, TLR4 |
| MIF-mediated Glucocorticoid Regulation | 0.45 | 2.02E-07 | -2.24 | 9.39E-08 | CD14, LY96, NFKB1, RELA, TLR4 |
| Osteoarthritis Pathway | -0.82 | 5.17E-05 | -2.24 | 2.82E-05 | CASP1, CXCL8, IL1R1, NFKB1, RELA, TLR4 |
| Type I Diabetes Mellitus Signaling | 0.38 | 8.04E-08 | -2.24 | 1.84E-05 | IL1R1, IRAK1, MYD88, NFKB1, RELA |
| IL-1 Signaling | 0.33 | 1.88E-11 | -2.12 | 1.94E-10 | FOS, IL1A, IL1R1, IRAK1, IRAK2, MYD88, NFKB1, RELA |
| p38 MAPK Signaling |  | 4.76E-02 | -2.00 | 4.29E-04 | IL1A, IL1R1, IRAK1, IRAK2 |
| IL-17A Signaling in Airway Cells | -0.82 | 3.05E-09 | -2.00 | 1.78E-06 | CXCL1, CXCL3, CXCL5, NFKB1, RELA |
| Inflammasome pathway | -1.00 | 1.54E-06 | -2.00 | 8.43E-07 | CASP1, MYD88, NFKB1, TLR4 |
| LXR/RXR Activation | 1.63 | 3.25E-06 | 3.00 | 5.54E-11 | CCL2, CCL7, CD14, IL1A, IL1R1, LY96, NFKB1, RELA, TLR4 |

Additional File 4. Apabetalone’s Gene Targets in IPA® Canonical Pathways Gene Sets: Unstimulated Control and DM2+CVD monocytes treated with apabetalone *ex vivo*.

*IPA® z-scores compare the observed differential regulation of a gene (“activating” or “inhibiting”) in the dataset to changes predicted by the literature. z<-2 predicts a downregulation and z>2 predicts an upregulation within a gene set associated with a canonical pathway or a transcriptional regulator.

‡p‐value<0.01 indicates a statistically significant overlap between the dataset genes and the curated gene sets (Fisher’s exact test).

#Genes expression changes >20%, p<0.05 as detected by Nanostring.

Additional File 5. Apabetalone’s Gene Targets That Converge on IPA® Upstream Regulators: Unstimulated Monocytes Treated with apabetalone *ex vivo*.

| **IPA®**  **Upstream Regulators** | **z-score***  **Controls**  **Apa vs. DMSO** | **p-value‡** | **z-score***  **DM2+CVD Apa vs. DMSO** | **p-value‡** | **Apabetalone Gene Targets in DM2+CVD Monocytes#** |
| --- | --- | --- | --- | --- | --- |
| TNF | -1.66 | 3.57E-23 | -3.90 | 4.76E-17 | C3AR1, CASP1, CCL2, CCL7, CCR1, CD14, CD163, CXCL1, CXCL2, CXCL3, CXCL5, CXCL8, CYBB, FCAR, FOS, FPR1, FPR2, IL1A, IL1R1, IL3RA, IRAK2, ITGAX, LY96, MYD88, NFKB1, RELA, THBS1, TLR4 |
| LPS | -1.09 | 1.43E-29 | -3.86 | 3.07E-24 | C3AR1, CASP1, CCL2, CCL7, CCR1, CD14, CD163, CD68, CXCL1, CXCL2, CXCL3, CXCL5, CXCL8, CYBB, FOS, FPR1, FPR2, IL10RB, IL1A, IL1R1, IL3RA, IRAK1, IRAK2, ITGAX, LY96, MARCO, MYD88, NFKB1, RELA, TBK1, THBS1, TLR1, TLR4,TLR6 |
| IFNG | -0.96 | 4.96E-23 | -3.34 | 9.71E-23 | CASP1, CCL2, CCL7, CCR1, CD14, CD163, CD68, CXCL1, CXCL2, CXCL3, CXCL5, CXCL8, CYBB, FCGR3A/FCGR3B, FOS, FPR2, IL1A, IL1R1, IL3RA, IRAK1, IRAK2, ITGAX, LY96, MYD88, NFKB1, RELA, THBS1, TLR1, TLR4, TLR6 |
| TLR4 | -1.26 | 2.05E-16 | -3.26 | 3.50E-10 | CCL2, CD14, CD163, CXCL2, CXCL3, CXCL8, IL1A, MARCO, MYD88, NFKB1, RELA, TLR4 |
| APP | -1.59 | 1.87E-09 | -3.15 | 5.51E-09 | CCL2, CCL7, CD68, CXCL1, CXCL2, CXCL3, CXCL5, CXCL8, CYBB, FOS, IL1A, MYD88, NFKB1, RELA, TLR4 |
| CSF2 | -1.50 | 2.02E-26 | -3.07 | 6.18E-25 | CASP1, CCL2, CCR1, CD14, CD163, CD180, CXCL1, CXCL2, CXCL8, CYBB, FOS, FPR2, IL1A, IL1R1, IL3RA, IRAK1, ITGAX, LY96, MARCO, NFKB1, RELA, THBS1, TLR1, TLR4 |
| IL17A | -2.30 | 1.89E-10 | -3.01 | 3.73E-12 | CCL2, CD14, CD163, CD68, CXCL1, CXCL2, CXCL3, CXCL5, CXCL8, FOS, IL1A, TLR4 |
| NFkB | -1.26 | 1.40E-15 | -3.00 | 4.43E-10 | CCL2, CCL7, CXCL1, CXCL2, CXCL3, CXCL5, CXCL8, CYBB, FOS, FPR2, IL1A, NFKB1, RELA, TLR4 |
| IL1B | -1.16 | 7.51E-25 | -2.97 | 1.18E-18 | CCL2, CCL7, CCR1, CD14, CXCL1, CXCL2, CXCL3, CXCL5, CXCL8, CYBB, FOS, FPR2, IL1A, IL1R1, IL3RA, IRAK1, IRAK2, LY96, MYD88, NAD+, NFKB1, RELA, THBS1, TLR4 |
| RELA | -1.24 | 2.65E-14 | -2.96 | 1.09E-09 | CCL2, CD14, CXCL1, CXCL2, CXCL3, CXCL5, CXCL8, CYBB, FOS, IL1A, NFKB1, RELA |
| IFNA | -1.16 | 1.14E-19 | -2.92 | 2.39E-14 | C3AR1, CASP1, CCL2, CCL7, CCR1, CXCL1, CXCL2, CXCL3, CXCL5, CXCL8, FOS, IL1R1, MYD88, RELA, TLR1, TLR4 |
| LDL | -1.95 | 4.89E-14 | -2.85 | 2.71E-10 | CASP1, CCL2, CD68, CXCL2, CXCL3, CXCL8, CYBB, FOS, IL1A, NFKB1, TLR4 |
| OSCAR | -1.67 | 3.74E-17 | -2.83 | 2.24E-13 | CCL7, CXCL1, CXCL2, CXCL3, CXCL5, CXCL8, IL1A, IL1R1 |
| HMGB1 | -2.41 | 5.00E-08 | -2.79 | 3.00E-10 | CCL2, CD163, CXCL3, CXCL5, CXCL8, IL1A, IL1R1, TLR4 |
| FN1 | -1.50 | 1.11E-15 | -2.78 | 7.42E-09 | CCL2, CXCL1, CXCL2, CXCL3, CXCL5, CXCL8, FOS, IL1R1, THBS1 |
| cholesterol | -1.73 | 6.89E-12 | -2.78 | 1.18E-13 | CASP1, CCL2, CD14, CD68, CXCL2, CXCL3, CXCL8, CYBB, IL10RB, IL1A, ITGAX, THBS1, TLR4 |
| TICAM1 | -1.29 | 8.55E-13 | -2.76 | 9.22E-09 | CCL2, CXCL2, CXCL3, CXCL8, FPR1, FPR2, IL1A, NFKB1 |
| MYD88 | -2.45 | 2.56E-17 | -2.76 | 1.59E-14 | CCL2, CD14, CXCL1, CXCL2, CXCL3, CXCL8, FOS, FPR1, FPR2, IL1A, ITGAX, MARCO, NFKB1, RELA |
| EGR1 | -2.40 | 4.02E-10 | -2.76 | 6.30E-08 | CCL2, CCR1, CXCL2, CXCL3, CXCL8, NFKB1, THBS1, TLR4 |

*IPA® z-scores compare the observed differential regulation of a gene (“activating” or “inhibiting”) in the dataset to changes predicted by the literature. z-score<-2 predicts a downregulation and z-score>2 predicts an upregulation within a gene set associated with a canonical pathway or a transcriptional regulator.

‡p‐value<0.01 indicates a statistically significant overlap between the dataset genes and the curated gene sets (Fisher’s exact test).

#Genes expression changes >20%, p<0.05 as detected by Nanostring.

Additional File 6. Apabetalone’s Gene Targets within IPA® Canonical Pathways: IFNγ stimulated monocytes treated with apabetalone *ex vivo*.

| **IPA®**  **Canonical Pathways** | **Cohort** | **z-score***  **IFNγ vs. DMSO** | **p-value‡** | **z-score***  **IFNγ+**  **Apa vs. IFNγ** | **p-value‡** | **Apabetalone Gene Targets in**  **Monocytes Stimulated with IFN** |
| --- | --- | --- | --- | --- | --- | --- |
| Role of PRRs in Recognition of Bacteria and Viruses | Control | 2.3 | <0.0001 | 1.0 | <0.0001 | C3AR1,IFIH1,IRF7,MAPK1,MAPK8,MYD88,NLRP3,OAS3,TLR1,TLR5,TLR8,TRAF6 |
|  | DM2+CVD | 2.7 | <0.0001 | -2.3 | <0.0001 | C3AR1,CASP1,CXCL8,DDX58,IFIH1,IL1A,MAPK8,MYD88,NFKB1,NLRP3,OAS3,RELA,RIPK2,TLR1,TLR4,TLR8,TNF |

*IPA® z-scores compare the observed differential regulation of a gene (“activating” or “inhibiting”) in the dataset to changes predicted by the literature. z-score<-2 predicts a downregulation and z-score>2 predicts an upregulation within a gene set associated with a canonical pathway or a transcriptional regulator.

‡p-value<0.01 indicates a statistically significant overlap between the dataset genes and the curated gene sets (Fisher’s exact test).

#Genes expression changes >20%, p<0.05 as detected by Nanostring.

Additional File 7. Apabetalone’s Gene Targets in IPA® Upstream Regulators Gene Sets: IFNγ stimulated control and DM2+CVD patient monocytes treated with apabetalone *ex vivo*.

|  | **z-score*** | | | |  |
| --- | --- | --- | --- | --- | --- |
| **Gene Symbol** | **CTL**  **IFNγ **  **vs. DMSO** | **CTL**  **IFNγ+Apa **  **vs. IFNγ** | **DM2+CVD IFNγ **  **vs. DMSO** | **DM2+CVD IFNγ+Apa vs. IFNγ** | **Apabetalone Gene Targets in DM2+CVD monocytes#** |
| IFNG | 4.49 | -2.15 | 4.85 | -4.72 | CASP1, CCL2, CCL3, CCL4, CCL7, CCL8, CCR1, CD14, CD163, CD68, CXCL1, CXCL10, CXCL2, CXCL8, CXCL9, CYBB, DDX58, FCGR3A/FCGR3B, FOS, FPR2, IDO1, IFIH1, IFITM1, IFITM2, IL1A, IL3RA, IRAK1, IRAK2, ITGAX, LY96, MSR1, MX1, MYD88, NFKB1, NLRP3, OAS3, RELA, RIPK2, STAT1, THBS1, TLR1, TLR4, TLR8, TNF |
| TNF | 1.79 | -0.85 | 2.66 | -4.20 | C3AR1, CASP1, CCL2, CCL3, CCL4, CCL7, CCR1, CD14, CD163, CXCL1, CXCL10, CXCL2, CXCL8, CXCL9, CYBB, DDX58, FCAR, FOS, FPR1, FPR2, IDO1, IFIH1, IFITM1, IL1A, IL3RA, IRAK2, ITGAX, LY96, MSR1, MX1, MYD88, NFKB1, NLRP3, OAS3, RELA, RIPK2, STAT1, THBS1, TLR4, TLR8, TNF |
| Interferon alpha | 3.88 | -2.15 | 3.95 | -4.01 | C3AR1, CASP1, CCL2, CCL3, CCL7, CCR1, CXCL1, CXCL10, CXCL2, CXCL8, CXCL9, DDX58, FOS, IDO1, IFIH1, IFITM1, IFITM2, MX1, MYD88, OAS3, RELA, STAT1, TLR1, TLR4, TLR8, TNF |
| TLR4 | 1.42 | -0.97 | 2.10 | -3.60 | CCL2, CCL3, CCL4, CCL8, CD14, CD163, CXCL10, CXCL2, CXCL8, IL1A, MX1, MYD88, NFKB1, RELA, RIPK2, STAT1, TLR4, TNF |
| IL1B | 1.73 | -1.27 | 2.66 | -3.52 | CCL2, CCL3, CCL4, CCL7, CCL8, CCR1, CD14, CXCL1, CXCL10, CXCL2, CXCL8, CXCL9, CYBB, FOS, FPR2, IDO1, IL1A, IL3RA, IRAK1, IRAK2, LY96, MX1, MYD88, NAD+, NFKB1, RELA, RIPK2, STAT1 ,THBS1, TLR4, TLR8, TNF |
| CSF2 | 1.28 | -1.79 | 2.19 | -3.48 | CASP1, CCL2, CCL3, CCL4, CCR1, CD14, CD163, CD180, CXCL1, CXCL10, CXCL2, CXCL8, CYBB, FOS, FPR2, HDAC3, IDO1, IL1A, IL3RA, IRAK1, ITGAX, LY96, NFKB1, NLRP3, RELA, RIPK2, THBS1, TLR1, TLR4, TNF |
| SPI1 | 1.99 | -0.78 | 2.54 | -3.41 | C3AR1, CCL3, CCL4, CD14, CD180, CD68, CXCL10, CYBB, FOS, IFITM1, MX1, RELA, TLR4, TNF |
| IFNA2 | 3.82 | -1.67 | 3.82 | -3.18 | CCL2, CCL3, CCL8, CXCL10, CXCL9, DDX58, IDO1, IFIH1 ,IFITM1, IFITM2, LILRB2, MX1, OAS3, STAT1, TNF |
| CD40LG | 1.97 | 0.43 | 2.41 | -2.76 | CASP1, CCL2, CCL3, CCL4, CCR1, CXCL1, CXCL10, CXCL2, CXCL8, FOS, IDO1, IFITM1, IL1A, IL3RA, MX1, NFKB1, RELA, STAT1, TNF |
| IRF7 | 4.01 | -0.97 | 4.01 | -2.68 | CCL8, CXCL10, CXCL9, DDX58, IDO1, IFIH1, IFITM1, IFITM2, ITGAX, MX1, OAS3, RIPK2, STAT1, TLR4, TLR8 |
| IFNL1 | 2.63 | -1.40 | 2.63 | -2.65 | CXCL10, CXCL8, CXCL9, DDX58, IFIH1, IFITM1, IFITM2, MX1, OAS3, STAT1 |
| STAT1 | 2.17 | -0.91 | 2.64 | -2.48 | CASP1, CCL2, CCL3, CCL4, CD14, CXCL10, CXCL2, CXCL8, CXCL9, FOS, IDO1, IFIH1, IFITM1, IFITM2, ITGAX, LY96, MX1, OAS3, STAT1, TLR4, TLR8, TNF |
| IFNB1 | 4.55 | -1.08 | 4.66 | -2.46 | CASP1, CCL2, CCL3, CCL4, CCL7, CD14, CXCL10, CXCL2, CXCL8, CXCL9, DDX58, FOS, IDO1, IFIH1 ,IFITM1, ITGAX, MX1, MYD88, RIPK2, STAT1, THBS1, TLR8, TNF |
| STAT2 | 2.43 | -0.09 | 2.43 | -2.39 | CXCL10, CXCL8, IFITM1, IFITM2, MX1, STAT1, TNF |
| IFN Beta | 3.60 | -1.19 | 3.72 | -2.30 | C3AR1, CCL3, CCL7, CXCL1, CXCL10, CXCL8, DDX58, IDO1, IFIH1, IFITM1, MX1, OAS3, STAT1, TNF |
| IL27 | 2.05 | -1.26 | 2.18 | -2.21 | CCL2, CCL3, CCL4, CCL7, CD14, CD163, CXCL10, CXCL8, CXCL9, FOS, IL1A, MX1, STAT1, TNF |
| IL5 | 1.95 | 0.13 | 2.16 | -2.16 | CCL2, CCL3, CCR1, CXCL8, IL3RA, SPN, TLR1, TNF |
| IRF5 | 2.20 | -0.09 | 2.20 | -2.02 | CCL3, CCL4, CXCL10, CXCL2, DDX58, IFIH1, STAT1, TNF |

*IPA® z-scores compare the observed differential regulation of a gene (“activating” or “inhibiting”) in the dataset to changes predicted by the literature. z-score<-2 predicts a downregulation and z-score>2 predicts an upregulation within a gene set associated with a canonical pathway or a transcriptional regulator.

#Genes expression changes >20%, p<0.05 as detected by Nanostring.
